# Identification and Construction of a Long Noncoding RNA Prognostic Risk Model for Stomach Adenocarcinoma Patients

**DOI:** 10.1155/2021/8895723

**Published:** 2021-02-24

**Authors:** Zhiqiang Zha, Peiling Zhang, Dailing Li, Guolong Liu, Lin Lu

**Affiliations:** ^1^Department of Medical Oncology, Guangzhou First People's Hospital, School of Medicine, South China University of Technology, Guangzhou Guangdong, China; ^2^Department of Medical Oncology, Guangzhou First People's Hospital, Guangzhou Medical University, Guangzhou, Guangdong, China 510180

## Abstract

**Background:**

Long noncoding RNA-based prognostic biomarkers have demonstrated great potential in the diagnosis and prognosis of cancer patients. However, systematic assessment of a multiple lncRNA-composed prognostic risk model is lacking in stomach adenocarcinoma (STAD). This study is aimed at constructing a lncRNA-based prognostic risk model for STAD patients.

**Methods:**

RNA sequencing data and clinical information of STAD patients were retrieved from The Cancer Genome Atlas (TCGA) database. Differentially expressed lncRNAs (DElncRNAs) were identified using the R software. Univariate and multivariate Cox regression analyses were performed to construct a prognostic risk model. The survival analysis, C-index, and receiver operating characteristic (ROC) curve were employed to assess the sensitivity and specificity of the model. The results were verified using the GEPIA online tool and our clinical samples. Pearson correlation coefficient analysis, Gene Ontology (GO), and Kyoto Encyclopedia of Genes and Genomes (KEGG) pathway enrichment were performed to indicate the potential biological functions of the selected lncRNA.

**Results:**

A total of 1917 DElncRNAs were identified from 343 cases of STAD tissues and 30 cases of noncancerous tissues. According to univariate and multivariable Cox regression analyses, four DElncRNAs (AC129507.1, LINC02407, AL022316.1, and AP000695.2) were selected to establish a prognostic risk model. There was a significant difference in the overall survival between high-risk patients and low-risk patients based on this risk model. The C-index of the model was 0.652. The area under the curve (AUC) for the ROC curve was 0.769. GEPIA results confirmed the expression and prognostic significance of AP000695.2 in STAD. Our clinical data confirmed that upregulated expression of AP000695.2 was correlated with the T stage, distant metastasis, and TNM stage in STAD. GO and KEGG analyses demonstrated that AP000695.2 was closely related to the tumorigenesis process.

**Conclusions:**

In this study, we constructed a lncRNA-based prognostic risk model for STAD patients. Our study will provide novel insight into the diagnosis and prognosis of STAD patients.

## 1. Introduction

Stomach adenocarcinoma (STAD), as the most common type of gastric cancer (GC), is characterized by rapid growth and strong invasiveness [1]. It is among one of the leading causes of cancer-related death worldwide [2, 3]. Approximately 70% of stomach adenocarcinoma was diagnosed in developing countries [3]. To date, due to the late stage of diagnosis and lack of effective treatment strategies, the prognosis of stomach adenocarcinoma is unsatisfactory [4, 5]. Moreover, the development of targeted therapy and immune checkpoint inhibitors only benefits a very small population of gastric cancer patients [6–8]. The main reason for these phenomena is the lack of effective diagnosis and prognostic evaluation measures for STAD at an early stage [9, 10]. Therefore, identifying novel biomarkers which could diagnose and predict the survival of STAD patients is critical.

Long noncoding RNA (lncRNA) belongs to noncoding RNA molecules, which are longer than 200 nucleotides (nt) at length [11]. lncRNAs were initially considered to have no physiological function but a byproduct of RNA polymerase II transcription [12]. However, due to the development of next-generation sequencing technologies, lncRNAs emerge as crucial regulators in tumorigenesis [13–15]. Recent studies revealed that lncRNAs participate in various biological processes including transcriptional regulation, RNA editing, and posttranscriptional regulation of many genes [14, 16, 17]. There were close correlations between lncRNAs and various cellular events, including cell proliferation, migration, invasion, cell cycle, and apoptosis [18–20]. With regard to gastric cancer, lncRNAs have been detected and could function as oncogenes or tumor suppressors [21, 22]. Dysregulated expression of lncRNAs demonstrated important roles in predicting the relapse, metastasis, chemoresistance, and overall survival of gastric cancer [20, 23]. Upregulated expression of lncRNA AC093818.1 demonstrated high sensitivity in predicting metastatic gastric cancer and could accelerate gastric cancer metastasis [20]. Cisplatin-resistant gastric cancer patients demonstrated higher lncRNA HOXD-AS1 expression [23]. lncRNA LOC100130476 was related to tumor suppression and aberrant methylation in gastric cardia adenocarcinoma [24]. lncRNA N-BLR could promote the migration and invasion of gastric adenocarcinoma [25]. In addition, lncRNAs RP11-169F17.1 and RP11-669N7.2 were regarded as prognostic biomarkers of stomach adenocarcinoma [26]. lncRNA RP1-163G9.1 was associated with poor prognosis of gastric adenocarcinoma patients [12]. All these studies indicate that lncRNAs could serve as prognostic biomarkers for gastric cancer patients. However, the specificity and sensitivity of single lncRNA as a biomarker are limited. An integrated lncRNA prognostic risk model would play more vital roles in the diagnosis and prognosis of STAD patients [12, 24–26].

In this study, we established a prognostic risk model by analyzing RNA sequencing data from TCGA dataset. The results were verified using the GEPIA online tool and our clinical data. This study will provide novel insight into the prognostic values of the lncRNA-based model, which could be used in tumor diagnosis and survival prognosis for STAD patients.

## 2. Material and Methods

### 2.1. Ethical Statement

A total of 78 STAD patients who underwent surgery between January 2010 and December 2017 were included in this study. None of them had received any antitumor therapies prior to the collection of the tissue samples. There were 25 females and 53 males, with a median age of 52 years (range, 21-75 years). All tumor tissues and adjacent normal tissues were obtained with informed consent, and the study was approved by the Research Ethics Committee of Guangzhou First People's Hospital, South China University of Technology.

### 2.2. Data Retrieval and Identification of Differently Expressed lncRNAs (DElncRNAs)

The RNA sequence data and corresponding clinical information from 343 STAD patients were downloaded from TCGA dataset (up to December 2019). There were 343 cases of tumor tissues and 30 cases of adjacent nontumoral tissues included in this study. The data was retrieved using the Perl software. The DElncRNAs were compared between tumor tissues and adjacent nontumoral tissues using the “edgeR” package based on the R language. DElncRNAs were identified using the following criteria: ∣logfold change | >1 and adjusted *P* value < 0.05. The heatmap was drawn using the “pheatmap” package in the R software.

### 2.3. Survival Analysis Using Kaplan-Meier Survival Analysis

The correlations between DElncRNAs and overall survival of STAD patients were analyzed using Kaplan-Meier survival analysis with the log-rank test in the R software, where *P* < 0.05 was considered statistically significant.

### 2.4. Cox Regression Analysis

Univariate and multivariate Cox regression analyses were performed to establish a prognostic risk model which could independently predict the survival of STAD patients. After being subjected to univariate Cox regression analysis, the DElncRNAs with *P* value < 0.001 were further subjected to a multivariate Cox proportional hazards model in the R software.

### 2.5. Assessment of the Prognostic Risk Model

Kaplan-Meier survival analysis was used to assess the survival curve in the high-risk and low-risk groups using the “survival” package in the R software. The C-index value of the prognostic risk model was calculated using the “survcomp” package. Receiver operating characteristic (ROC) curve analysis was performed using the “survivalROC” package in the R software.

### 2.6. Validation of the Expression and Prognostic Values of the Independent Prognostic lncRNAs

The expression and prognostic significance of these independent prognostic lncRNAs were further verified using the online tool Gene Expression Profiling Interactive Analysis (GEPIA, http://http://gepia.cancer-pku.cn/index.html).

### 2.7. Real-Time Quantitative PCR (qRT-PCR)

Total RNAs were collected from 78 paired STAD tumor tissues and adjacent normal tissues and extracted by the TRIzol reagent (Invitrogen, USA). The synthesis of cDNA was conducted by using HiScript II 1st Strand cDNA Synthesis Kit (Vazyme Biotech, Nanjing, China) from isolated RNA [27, 28]. The cDNA was subjected to the real-time quantitative PCR using the SYBR Green Master Mix. GAPDH and 18S were used as internal controls. The sequences of the sense and antisense primers were as follows: 5′-GGACACTCTGAAGGAACTC-3′ (F) and 5′-GATGACCATTAGCCAACAAG-3′ (R) for AP000695.2; 5′-CTCCTCCTGTTCGACAGTCAGC-3′ (F) and 5′-CCCAATACGACCAAATCCGTT-3′ (R) for GAPDH; and 5′-CGGCGACGACCCATTCGAAC-3′ (F) and 5′-GAATCGAACCCTGATTCCCCGTC-3′ (R) for 18S. The comparative threshold cycle (2^−ΔΔ^CT) method was used to calculate the relative mRNA values. The relative expression levels of AP000695.2 were normalized to the value of GAPDH.

### 2.8. Correlation between lncRNA and Coexpressed Protein Coding Genes

The correlations between lncRNAs and their coexpressed core genes were assessed using the “limma” package in the R software. The results were considered statistically significant when the Pearson correlation coefficient was greater than 0.4 and *P* value < 0.05.

### 2.9. Gene Oncology (GO) Annotation and KEGG Pathway Enrichment Analysis

GO enrichment analysis and KEGG pathway enrichment were performed using the “clusterProfiler” package in the R software. A *P* value < 0.05 was considered statistically significant.

### 2.10. Statistical Analysis

Data are presented as mean ± SD. The statistical analyses were assessed using the GraphPad Prism software (version 7.0). Two-tailed Student's *t*-test was used to assess the statistical differences between the two groups. The chi-square test or Fisher's exact test was used to compare the differences between AP000695.2 expression and clinical parameters. A two-tailed *P* value < 0.05 was considered statistically significant.

## 3. Results

### 3.1. Identification of Differentially Expressed lncRNAs (DElncRNAs) in STAD Patients

**Figure 1:**
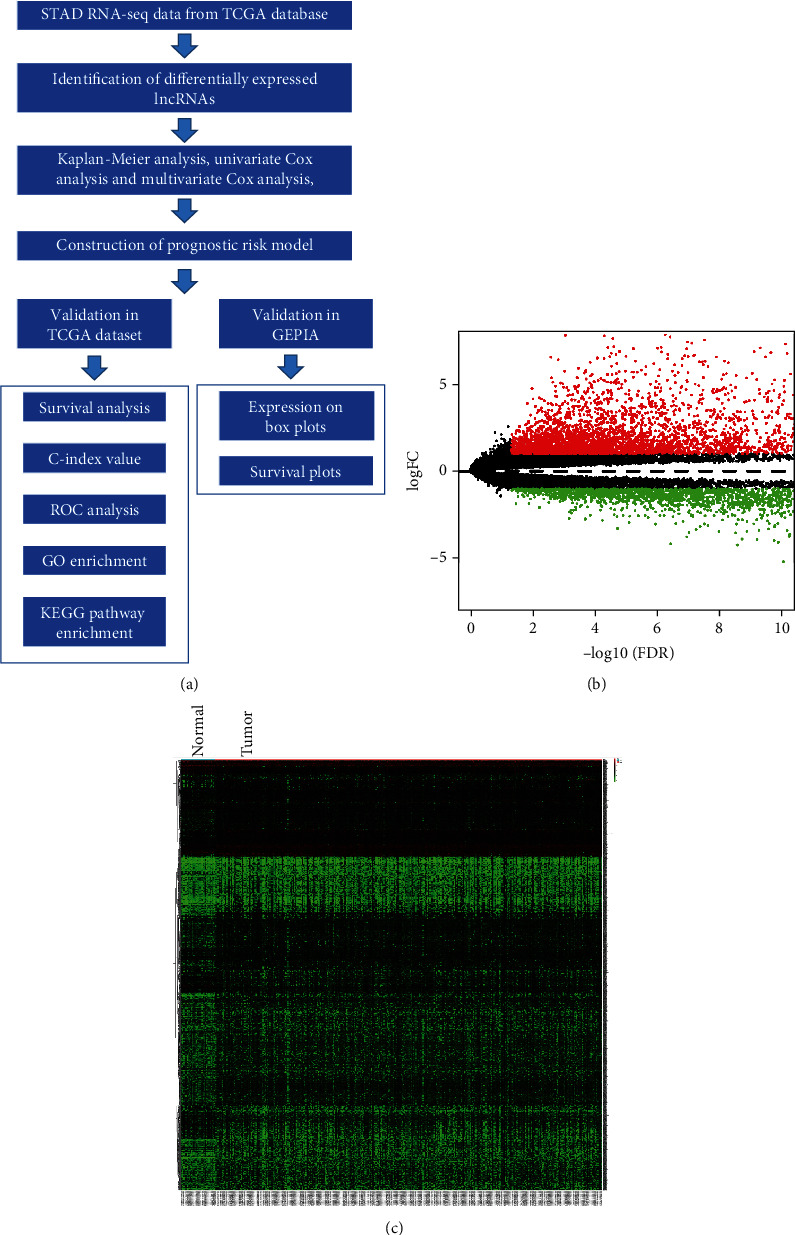
Identification of differentially expressed lncRNAs (DElncRNAs) in stomach adenocarcinoma patients. (a) The main flowchart of the current study. The data was retrieved from TCGA database. Differentially expressed lncRNAs were compared between 343 stomach adenocarcinoma tissues and 30 adjacent nontumoral tissues. (b) Volcano map of DElncRNAs. Red plots demonstrate upregulated lncRNAs, while green plots demonstrate downregulated lncRNAs. (c) Heatmap of the DElncRNAs between tumor tissues and adjacent nontumoral tissues. Red, black, and green colors represent the relatively high, medium, and low expression of lncRNAs, respectively. STAD: stomach adenocarcinoma; TCGA: The Cancer Genome Atlas; ROC: receiver operating characteristic curve; GO: Gene Ontology; KEGG: Kyoto Encyclopedia of Genes and Genomes; GEPIA: Gene Expression Profiling Interactive Analysis.

The overview of the screening strategy is shown in Figure 1(a). The identification of DElncRNAs was compared between 343 cases of tumor tissues and 30 cases of adjacent normal tissues based on TCGA database. Volcano plots demonstrated the distribution of DElncRNAs in STAD patients (Figure 1(b)). A total of 1917 DElncRNAs were identified, including 438 downregulated and 1497 upregulated lncRNAs.  Figure 1(c) shows the heatmap of the DElncRNAs compared between STAD tumor tissues and adjacent nontumoral tissues.

### 3.2. Construction of a Prognostic Risk Model in STAD Patients

**Figure 2:**
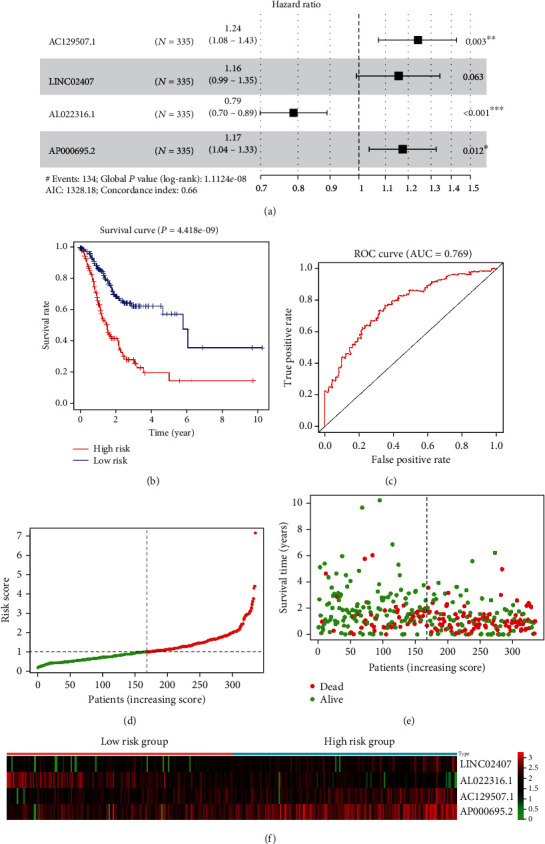
Construction and performance of a prognostic risk model. (a) Multivariate Cox regression analysis and forest plots demonstrate that key DElncRNAs could be viewed as independent prognostic biomarkers. (b) Kaplan-Meier curves demonstrate the difference between the high-risk group and low-risk group in OS based on the prognostic risk model. (c) ROC analysis of the prognostic risk model. (d) Scatter diagram shows the distributions of risk scores of STAD patients. (e) Scatter diagram shows the survival status of the patients based on this prognostic risk model. (f) Heatmap of the 4 prognostic lncRNA expression profiles.

To study the potential values of DElncRNAs in predicting the overall survival of STAD patients, we constructed a prognostic risk model based on the univariate and multivariate COX analysis. The univariate analysis was performed to identify possible diagnostic and prognostic biomarkers for STAD patients.  Table 1 shows the identified DElncRNAs with *P* value < 0.001. With a standard of *P* value < 0.001, 7 DElncRNAs (AL022316.1, AC129507.1, AP000695.1, FLJ16779, LINC01537, LINC02407, and AP000695.2) were subjected to further multivariate Cox regression analysis. We constructed a prognostic risk model containing 4 DElncRNAs (AC129507.1, LINC02407, AL022316.1, and AP000695.2). The forest plots demonstrated that AC129507.1 (*P* = 0.003), AL022316.1 (*P* < 0.001), and AP000695.2 (*P* = 0.012) could be used as independent biomarkers for STAD patients (Figure 2(a)).

We next assessed the sensitivity and specificity of this risk model. Based on the prognostic risk model, patients could be divided into the high-risk group and low-risk group. As shown in Figure 2(b), the patients classified into the high-risk group demonstrated a poorer survival rate than patients in the low-risk group (*P* = 4.418*e* − 09). The C-index of this risk model is 0.652 (95% CI, 0.606-0.698, Table 2). The ROC was calculated, and the value of the AUC is 0.769 (Figure 2(c)). All these data indicated that this prognostic risk model demonstrated good sensitivity and specificity.  Figure 2(d) shows the risk score distribution of the STAD patients.  Figure 2(e) shows the survival status of the STAD patients based on the risk model. The heatmap shows the lncRNA expression profiles of the prognostic risk model based on the risk score (Figure 2(f)).

### 3.3. Expression and Prognostic Significance of the DElncRNAs in the Prognostic Risk Model Based on TCGA Database

**Figure 3:**
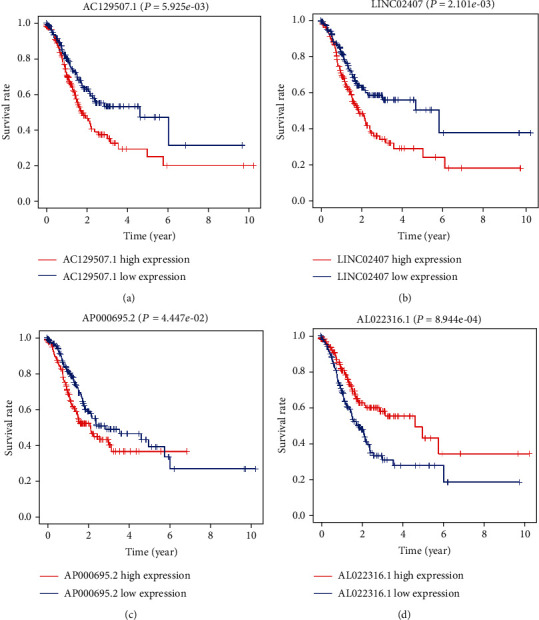
The correlations between prognostic lncRNAs and overall survival of STAD patients. Kaplan-Meier curves with the log-rank test for AC129507.1 (a), LINC02407 (b), AP000695.2 (c), and AL022316.1 (d).

The different expression levels of the independent prognostic lncRNAs are summarized in Table 3. Survival analysis for the independent prognostic lncRNAs was calculated using Kaplan-Meier survival analysis. As shown in Figure 3, upregulated expression of AC129507.1 (*P* = 5.925*e* − 09, Figure 3(a)), LINC02407 (*P* = 2.101*e* − 03, Figure 3(b)), and AP000695.2 (*P* = 4.447*e* − 02, Figure 3(c)) was positively correlated with poor overall survival, while high expression of AL022316.1 (*P* = 8.944*e* − 04, Figure 3(d)) was positively correlated with better OS.

### 3.4. Expression and Prognostic Values of the DElncRNAs in the Prognostic Risk Model Using GEPIA

**Figure 4:**
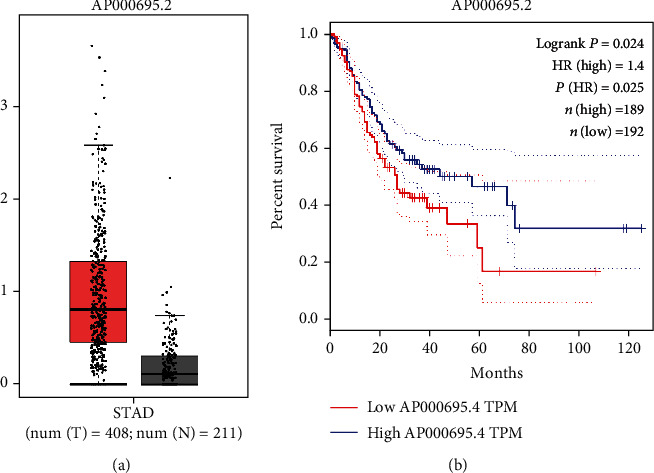
The expression and prognostic value of AP000695.2 were analyzed by GEPIA. There were 408 tumor samples and 211 normal samples in the GEPIA database. (a) The expression of AP000695.2 in STAD tumor tissues and normal tissues. (b) Kaplan-Meier survival curve for AP000695.2.

The online tool GEPIA was conducted to verify the expression and prognostic values of the prognostic DElncRNAs. A total of 408 tumor samples and 211 normal samples were included in GEPIA. As shown in Figure 4(a), upregulated expression of AP000695.2 was detected in the STAD tumor tissues. These data were in accordance with the data retrieved from TCGA database (Table 3). Overall survival analyses were also in accordance with the results of TCGA database. As shown in Figure 4(b), the high AP000695.2 expression (*P* = 0.024, Figure 4(b)) was positively correlated with poorer OS.

### 3.5. Validation of the Expression and Prognostic Value of AP000695.2 in STAD Patients

**Figure 5:**
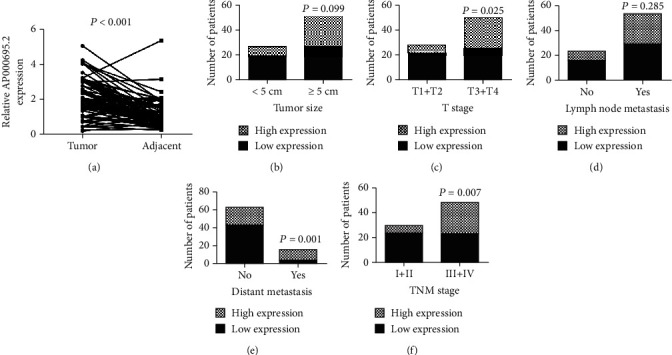
Validation of AP000695.2 expression and prognostic value in STAD patients. (a) The mRNA expression level of AP000695.2 in 78 paired STAD tumor tissues and adjacent normal tissues using real-time quantitative PCR (qRT-PCR). GAPDH was used as an endogenous control. The correlations between AP000695.2 expression and tumor size (b), T stage (c), lymph node metastasis (d), metastasis (e), and TNM stage (f) were analyzed.

The expression of AP000695.2 in 78 paired STAD tumor tissues and adjacent normal tissues was compared using real-time quantitative PCR (qRT-PCR). As shown in Figure 5(a), GAPDH was used as an endogenous control and the results demonstrated that the relative mRNA level of AP000695.2 was much higher in tumor tissues than in normal tissues (*P* < 0.001). Application of 18S as another internal control also confirmed the upregulated expression of AP000695.2 in STAD tumor tissues (*P* < 0.001, Supplementary Figure 1). The mean relative expression level of AP000695.2 was 2.141 (range, 0.19-5.04). The patients were divided into two groups based on the mean mRNA value of AP000695.2: low-expression group (*N* = 45) and high-expression group (*N* = 33). Moreover, the correlations between AP000695.2 expression and clinicopathological parameters were assessed (Figures 5(b)–5(f)). Upregulated expression of AP000695.2 was positively correlated with the T stage (*P* = 0.025, Figure 5(c)), distant metastasis (*P* = 0.001, Figure 5(e)), and TNM stage (*P* = 0.007, Figure 5(f)). However, there was no statistical significance between AP000695.2 expression and tumor size (*P* = 0.099, Figure 5(b)) and lymph node metastasis (*P* = 0.285, Figure 5(d)). Taken together, TCGA database, GEPIA online database, and our clinical samples indicated that lncRNA AP000695.2 might work as an oncogene in STAD.

### 3.6. Coexpressed Genes of AP000695.2

**Figure 6:**
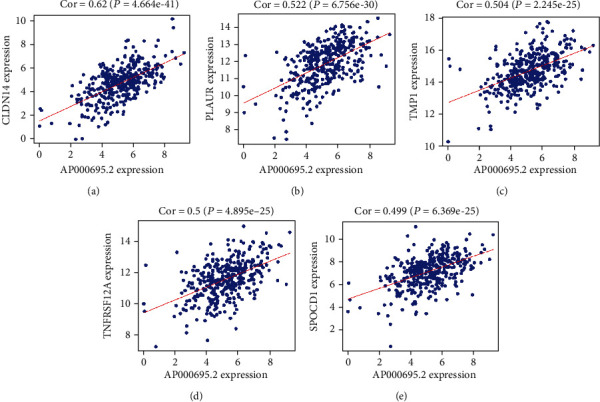
The correlations between AP000695.2 and its coexpressed genes. The correlation plots of top 5 correlated mRNAs of AP000695. Pearson correlation coefficient > 0.4 and *P* < 0.01.

We further studied the coexpressed protein coding genes of AP000695.2 to clarify its potential biological functions. According to the cut-off criteria (Pearson correlation coefficient > 0.4 or <-0.4 and *P* value < 0.001), AP000695.2 coexpressed with 59 mRNAs.  Figure 6 shows the top 5 correlating mRNAs of AP000695.2. AP000695.2 expression was positively correlated with CLDN14 (Figure 6(a)), PLAUR (Figure 6(b)), TMP1 (Figure 6(c)), TNFRSF12A (Figure 6(d)), and SPOCD1 (Figure 6(e)).

### 3.7. GO and KEGG Pathway Enrichment Analysis of the Independent Prognostic DElncRNAs

**Figure 7:**
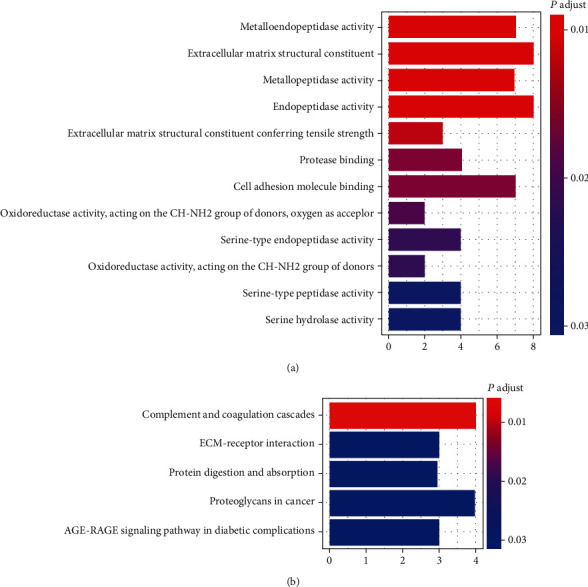
Functional enrichment analysis for AP000695.2 coexpressed protein coding genes: (a) bubble chart of GO enrichment and (b) bubble chart of KEGG pathway enrichment.

To further clarify the potential biological functions of AP000695.2 in STAD, we analyzed GO and KEGG pathway enrichment analysis. As shown in Figure 7(a), the GO enrichment results demonstrated obvious correlation with “extracellular matrix structural constituent,” “endopeptidase activity,” “metalloendopeptidase activity,” “metallopeptidase activity,” and “cell adhesion molecule binding” for AP000695.2. KEGG pathway enrichment analysis demonstrated that these genes were correlated with “complement and coagulation cascades,” “proteoglycans in cancer,” “ECM-receptor interaction,” “protein digestion and adsorption,” and “AGE-RAGE signaling pathway in diabetic complications” pathways (Figure 7(b)). All these data suggested that AP000695.2 was involved in multiple cellular processes, including the processes of tumor development.

## 4. Discussion

Most of the stomach adenocarcinoma patients were diagnosed at an advanced stage. Thus, the treatment strategies for most of these patients were limited [29]. The advancement in targeted therapies and immunotherapies failed to demonstrate promising results in STAD patients [30, 31]. And they are still lacking molecular drugs that can specifically target STAD. With the development of the next-generation sequence, studies about noncoding RNAs have greatly accelerated [32, 33]. The development of RNA sequencing technology has facilitated the identification of novel lncRNAs [34]. A bunch of lncRNAs have been reported to play important roles in gastric cancer cell proliferation, invasion, and metastasis [35, 36]. Dysregulation of lncRNAs has been reported to be correlated with tumorigenesis and progression of stomach adenocarcinoma. Moreover, the expression and biological functions of lncRNAs in STAD have been widely explored [37].

Nowadays, lncRNA-based biomarkers have been widely used in the diagnosis and prognosis of gastric cancer patients [38, 39]. lncRNA SNHG15 demonstrated a prospective biomarker for the diagnosis and therapeutics for gastric cancer patients [39]. lncRNA MAGI2-AS3 was identified as epithelial-mesenchymal transition- (EMT-) related lncRNA, which could predict the progression of gastric cancer patients [38]. Moreover, some lncRNAs could be detected in plasma and serve as noninvasive biomarkers, which makes lncRNA an ideal biomarker for cancer patients [40]. Zheng et al. demonstrated that serum lncRNAs FAM49B-AS, GUSBP11, and CTDHUT could be viewed as biomarkers for gastric cancer patients [40]. In this study, we identified 1917 differentially expressed lncRNAs between STAD tumor tissues and adjacent nontumoral tissues. Then, we constructed a four lncRNA- (AC129507.1, LINC02407, AL022316.1, and AP000695.2) based prognostic risk model based on these DElncRNAs. This prognostic risk model demonstrated good sensitivity and specificity in predicting the risk and survival of STAD patients. According to this model, our clinical data also confirmed that upregulated expression of independent prognostic lncRNA AP000695.2 was positively correlated with the T stage, M stage, and TNM stage. All these data indicated that AP000695.2 might work as an oncogenic gene in STAD patients. However, there were no articles concerning the expression and the biological functions of AP000695.2 in cancer patients.

lncRNAs work in multiple ways to regulate the expression and functions of target genes, including signal, decoy, guide, and scaffold [41, 42]. According to the results of GO enrichment and KEGG pathway enrichment, AP000695.2 coexpressed genes were enriched in biological functions and pathways including extracellular matrix structural constituent, ECM-receptor interaction, and proteoglycans in cancer. All these data indicated that lncRNA AP000695.2 might be involved in the cellular process of tumorigenesis. Thus, further studies are needed to elucidate the biological functions and molecular mechanisms of AP000695.2 in STAD.

## 5. Conclusion

In conclusion, DElncRNAs significantly associated with oncogenesis and prognosis of STAD were identified from TCGA database. The independent prognostic lncRNAs demonstrated good sensitivity and specificity in predicting the survival of STAD patients. Moreover, our preliminary studies revealed that upregulated expression of AP000695.2 was correlated with poor overall survival and the biological functions of AP000695.2 were predicted. However, there are no researchers once clarifying the functions and mechanisms of these DElncRNAs. Our study provides a novel lncRNA-based prognostic risk model and a new direction for future studies, where we will further explore the biological functions and mechanisms of these lncRNAs in STAD. Meanwhile, we will detect the expression of these DElncRNAs using our own clinical samples to further confirm the sensitivity and specificity of these DElncRNAs in predicting the survival of STAD patients.

## Figures and Tables

**Table 1:** Univariate analysis results of the statistically significant lncRNAs (*P* < 0.001).

Gene	HR	*Z*	*P* value
AL022316.1	0.79131398	-3.91180144	9.16*E* − 05
AC129507.1	1.31492429	3.70563892	0.000210859
AP000695.1	1.28943052	3.51995878	0.000431614
FLJ16779	1.15788429	3.50127829	0.000463032
LINC01537	1.24779825	3.46898743	0.000522424
LINC02407	1.27355052	3.30854576	0.000937819
AP000695.2	1.23639943	3.30519073	0.000949118

**Table 2:** C-index analysis of the prognostic risk model.

C-index	SE	Lower	Upper	*P* value
0.6518902	0.02357405	0.6056859	0.6980945	1.1705*E* − 10

**Table 3:** Expression of DElncRNAs in the prognostic risk model based on TCGA database.

DElncRNAs	logFC	*P* value	FDR
AC129507.1	-1.66040229	1.40*E* − 18	4.35E-17
LINC02407	2.69475885	7.73*E* − 15	1.40*E* − 13
AL022316.1	2.04469210	2.04*E* − 08	1.34*E* − 07
AP000695.2	1.59480729	1.45*E* − 07	8.08*E* − 07

## Data Availability

All data in our study are available from the corresponding authors upon reasonable request.

## Abbreviations

STAD: Stomach adenocarcinoma
TCGA: The Cancer Genome Atlas
DElncRNAs: Differentially expressed lncRNAs
ROC: Receiver operating characteristic curve
GO: Gene Ontology
KEGG: Kyoto Encyclopedia of Genes and Genomes
lncRNA: Long noncoding RNA
GC: Gastric cancer
CLDN14: Claudin 14
PLAUR: Plasminogen activator urokinase receptor
TMP1: Thymidylate synthase gene
TNFRSF12A: Tumor necrosis factor receptor superfamily member 12A
SPOCD1: Spen paralogue and orthologue C-terminal domain containing 1.

## Supplementary Materials

Supplementary Materials Supplementary Figure 1: the mRNA expression level of AP000695.2 in 78 paired STAD tumor tissues and adjacent normal tissues using real-time quantitative PCR (qRT-PCR). 18S was used as an endogenous control.
