# Supplementary figures and images for: Identification and Construction of a Long Noncoding RNA Prognostic Risk Model for Stomach Adenocarcinoma Patients
Source: Dis Markers. 2021 Feb 24;2021:8895723. doi: 10.1155/2021/8895723 (PMC7929674; doi:10.1155/2021/8895723)

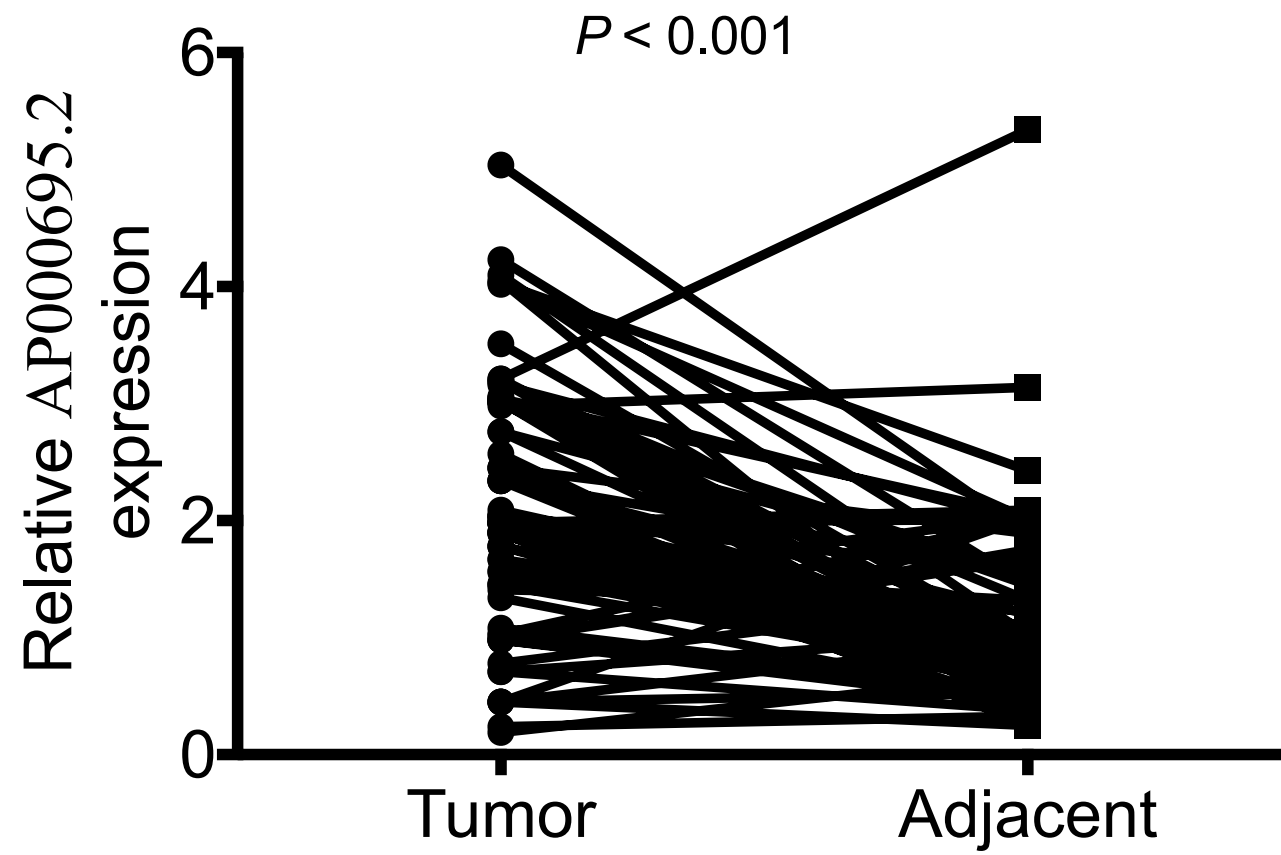

Supplement: Supplementary Materials — Supplementary Figure 1: the mRNA expression level of AP000695.2 in 78 paired STAD tumor tissues and adjacent normal tissues using real-time quantitative PCR (qRT-PCR). 18S was used as an endogenous control. [file 8895723.f1.pdf]
